
JOURNAL INFORMATION
==============================
NLM Title Abbreviation: Atlanta Med Surg J (1884)
NLM Journal ID: 101251623

Atlanta Medical and Surgical Journal (1884)

Publisher: Biomedical Journal Digitization Project

ARTICLE INFORMATION
==============================
PMCID: PMC9199299
PMID: 35829595
Article version: 1
Subjects: Original Communications

Report of Cases of Asthma Due to Intranasal Pressure

Crichton Logan M. M.D. Atlanta, Ga.

Print publication date: 1898 Dec
Volume: 15
Volume Series: New Ser
Issue: 10
First page: 669
Last page: 673
License: This work is free of known copyright restrictions. For more information, please see PMC Back Issue Digitization.
License URL: https://creativecommons.org/publicdomain/mark/1.0/

==============================

### REPORT OF CASES OF ASTHMA DUE TO INTRA-NASAL PRESSURE.

By LOGAN M. CRICHTON, M.D.,

Atlanta, Ga.

I have selected from my record of asthma patients four cases to present for your consideration this evening; two of these are complete, radical cures; two are incomplete, the last two, I think, being the most interesting and instructive.

While I have been making asthma and some other nasal reflexes a somewhat special study for the past few years, and have been deeply interested in them, I have tried to keep off of a hobby. I have not lost sight of the fact that many, a great many cases of asthma have nothing whatever to do with the nose. I have endeavored to keep clearly defined in my mind the conditions in the nasal chambers which would be likely to cause this distressing disease.

Many cases have applied to me for treatment, in whom I did not find these conditions; these cases I have refused to treat, and have referred them to their family physicians, believing the peripheral irritation to be elsewhere than the nose.

These conditions, in all the cases I have handled, have been clear and unmistakable; they should be easily recognized by the merest tyro in nasal surgery; they consist in malformations of the nasal septum or intra-nasal growths causing pressure. Many malformations, although they may produce a certain amount of obstruction to nasal respiration, do not cause any reflex trouble; the removal of such malformations will be followed by no results, so far as the reflexes are concerned.

It has been my observation that deformities of the septum deflections, exostoses, etc., are more prone to cause reflexes than the simple fleshy enlargements, such as hypertrophied turbinates, polypi, etc.

Case 1.—J. H., boy, aged fourteen years. Referred by Dr. J. A. Childs. Patient tall, for his age, perfectly healthy, although extremely thin. Has suffered from asthma since childhood. The attacks for several years becoming more severe, so distressing in fact, that many nights sleep in the recumbent posture was impossible. His only rest was obtained by kneeling at the side of the bed and resting his head on a pillow.

On making a careful examination I found my patient had true hypertrophy of the middle and inferior turbinate bodies of both sides, the bodies pressing tight against each other, as well as against the septum.

To the uninitiated I would like to explain that we have three forms of hypertrophy of the turbinates—the false, the beginning stages of the true, and the true. We apply a four per cent. solution of cocain to the turbinate in question, and wait ten minutes. If the cocain produces complete collapse of the turbinate, we have a false hypertrophy; if only partial, the beginning true; if to no appreciable degree, the hypertrophy is considered true.

Finding the hypertrophies in this case to be true, I advised operation.

I reduced the turbinates completely with the galvanocautery, and ordered a soothing, antiseptic spray.

Patient was discharged four years since and has not had an attack of asthma during the four years.

CASE 2.—Mr. R., aged twenty-one years. Referred by Dr. William Owens (at that time a medical student).

Mr. R.'s general condition was excellent; no trouble except asthma, from which he had suffered for ten years or more; was forced to spend several winters in Florida, where he was partially free from attacks. The seizures were violent, distressing and of long duration. Made a careful examination of the nasal chambers and found hypertrophy (true) of the middle and inferior turbinates on left side. On the right side I found a large bony cartilaginous projection from the septum, extending across the lumen of the nasal chamber and pressing firmly into the sulcus between the middle and inferior turbinate bodies. At point of contact the mucous membrane was exquisitely sensitive.

This was such a beautifully marked case that I promised the patient a cure, something I seldom do.

I first reduced the hypertrophied turbinates with the galvano-cautery. I removed the bony cartilaginous projection with the saw and chisel, meeting with considerable hemorrhage, on account of the large size of the growth.

I discharged patient about two years since. He has had absolutely no return of the asthma.

Case 3.—Mrs. M., aged about thirty-six. Referred by Dr. W. S. Elkin.

Patient a large, vigorous woman, absolutely healthy with the exception of being a great sufferer from asthma. The attacks were especially violent in the autumn, many nights sleep in the recumbent posture being impossible. Patient had taken all the remedies in the catalogue, with only temporary relief; was forced to seek the high altitude of Roan Mountain, where she was partially comfortable.

I found, on examination, true hypertrophy of the middle and inferior turbinate bodies on left side. On the right side a bony-cartilaginous projection from the septum, extending extirely across the lumen of the nostril and pressing tightly between the middle and inferior turbinates; the mucous membrane at point of contact was exquisitely sensitive.

I attacked the turbinates first, as is my custom, reducing them sufficiently to relieve all pressure and to give perfect breathing space. I then removed the bony cartilaginous projection from the septum with the nasal saw and chisel.

These operations were performed in July, August and September, 1895. The result of the work seemed all that any one could desire ; patient had absolutely no indications of asthma for nearly two years, and here the extremely interesting and instructive record begins : In the fall of 1897 patient came to my office much distressed and worried, saying she had suffered from an attack of asthma the night previous.

Examined the nasal chambers for some point of pressure, to account for this unexpected return of the old trouble. Found both nasal chambers seemingly in perfect condition ; tilting the head back, I brought into view the upper middle turbinate area ; here I found polypoid degeneration of the turbinate body, causing the body to press tightly against the septum. Thinking this sufficient cause for the asthmatic attack, I thoroughly removed the enlargement with the galvanocautery. Patient had several slight manifestations of asthma during the following two or three days; they then ceased entirely. I did not see patient again for one year; she then presented herself, reporting an attack. Examination revealed an hypertrophy of the middle turbinate, immediately below the site of the operation of the year before. I used the galvanocautery at this point and patient had no manifestation of asthma for a week; she then returned, reporting an attack the night before. Examination showed that I had not removed the hypertrophy sufficiently and that there was still marked pressure between the middle turbinate and the septum. I cauterized very thoroughly at this point, shrinking the turbinate clearly away from the septum. Patient had several slight attacks after this, due to the swelling at point of operation. Since that time she has had no return whatever.

This case to me is one of the clearest of cause and effect that I have ever handled.

CASE 4.—Mrs. B., aged twenty-five years. Brought to me by her husband, a student in the Atlanta Medical College. Patient extremely thin, with a distressed look about the face. Patient had been subject to asthmatic attacks for some seven or eight years. Had lost flesh, from one hundred and fifty pound to ninety-eight pounds. The seizures for the past few years had heen extremely severe and of almost daily occurrence. All the asthma remedies were tried; none seemed to give relief except morphia, so morphia was used liberally, with the usual unhappy result.

Examination revealed almost identically the same condition found in case 3; namely, true hypertrophy of the middle and inferior turbinates on left side and a bony cartilaginous projection from the septum, passing across the lumen of the nasal chamber and pressing firmly against the lower margin of the middle turbinate body.

I reduced both turbinates, fully relieving all pressure and giving free breathing space, then removed the bony cartilaginous growth with saw and chisel. The asthmatic attacks ceased almost entirely. Patient lived out of the city, and had to come in twice weekly to my office. During these visits she would invariably buy morphia. Her husband stated the case to me and decided that, as she was practically cured, he thought it best to stop her visits to the city ; in this I agreed with him.

Fourteen months after this patient came to my office and reported that she had had only three or four slight attacks since the last time she had seen me. She had improved very much in general condition and had gained twenty-three pounds in weight.

I will say, in conclusion, that although I do not believe all cases of asthma are due to intranasal pressure, I believe a great many are, and that in a disease as intractable and as difficult to handle as this, the nose should invariably be investigated and examined with great care.

---
